# The SLC41 Family of Magnesium Transporters: Molecular Regulators of Magnesium Homeostasis and Their Multifaceted Roles in Human Diseases

**DOI:** 10.3390/ijms27041673

**Published:** 2026-02-09

**Authors:** Yu Cao, Caijun Rao, Zhipeng Du

**Affiliations:** 1 Department of Gastroenterology, Institute of Liver and Gastrointestinal Diseases, Tongji Hospital, Tongji Medical College, Huazhong University of Science and Technology, Wuhan 430000, China; u202213523@hust.edu.cn; 2 The Second Clinical College, Tongji Medical College, Huazhong University of Science and Technology, Wuhan 430000, China; 3 Department of Geriatrics, Tongji Hospital, Tongji Medical College, Huazhong University of Science and Technology, Wuhan 430000, China

**Keywords:** SLC41, Mg^2+^ transporter, biomarkers, cancer, systemic diseases

## Abstract

Magnesium ion (Mg^2+^), particularly its free intracellular form, is indispensable for regulating diverse cellular functions. This critical role implies the existence of dedicated transporters and channels in the plasma membrane that coordinate Mg^2+^ uptake, intracellular storage, and efflux to maintain homeostasis. Although numerous molecular entities responsible for such Mg^2+^ transport have been reported over the past decades, there is still limited knowledge of their precise functions and disease implications. This review focuses on the solute carrier family 41 (SLC41), which consists of three isoforms (A1, A2, and A3) that share homology with the prokaryotic magnesium transporter E (MgtE) Mg^2+^ transporter family. Accumulating evidence has established SLC41A1 as the Na^+^/Mg^2+^ exchanger—a predominant Mg^2+^-efflux system. By contrast, the subcellular site of SLC41A2-mediated Mg^2+^ flux remains undefined, with potential roles at either the plasma membrane or organellar membranes, and SLC41A3 facilitates Na^+^-dependent Mg^2+^ efflux from mitochondria. Additionally, several studies have reported the association between SLC41s and diseases, including Parkinson’s disease, hepatocellular carcinoma, and nephronophthisis-related ciliopathies. By synthesizing current knowledge, this review aims to enhance the understanding of SLC41 transporters in health and disease and to explore their potential as therapeutic targets for clinical intervention.

## 1. Introduction

Magnesium (Mg^2+^), particularly its free intracellular form, plays an indispensable role in the brain, heart, bones, and skeletal muscle, among other tissues. It is involved in nearly all fundamental cellular processes, including protein synthesis, nucleic acid stability, enzymatic activities, bioenergetic metabolism, and neuromuscular excitability [1,2]. However, as the majority is bound to proteins and polynucleotides, the free cytosolic concentration is maintained between 0.5 and 1.2 mM—a level comparable to that in the extracellular fluid [3]. Consequently, the maintenance of Mg^2+^ homeostasis is dependent on the coordinated regulation of uptake, intracellular storage, and efflux through specific transporters and channels. It is established that Mg^2+^ is transported into and out of intracellular organelle compartments, and there are likely dedicated active plasma membrane Mg^2+^ transporters and channels for each step. Over the past decades, numerous molecular entities responsible for such Mg^2+^ transport have been reported [4,5].

The first Mg^2+^ transporters to be characterized in humans were the transient receptor potential cation channel subfamily M members 6 and 7 (TRPM6 and TRPM7) [6,7,8]. Other Mg^2+^ transporters include the solute carrier family 41 (SLC41) members 1, 2, and 3, claudin family members 16 and 19, nonimprinted in Prader–Willi/Angelman syndrome (NIPA) Mg^2+^ transporters 1 and 2, cyclin and CBS domain divalent metal cation transport mediators 1 through 4 (CNNM1 through CNNM4), magnesium transporter 1 (MagT1), and the magnesium-selective mitochondrial RNA splicing 2 (MRS2) [9,10].

This review aims to describe some of the recent insights into the identification and functions of SLC41, which consists of three isoforms (A1, A2, and A3) with recognized homology to the prokaryotic MgtE Mg^2+^ transporter family (Table 1) [11,12]. SLC41 transporters have been implicated in mediating Mg^2+^ influx and efflux across the plasma membranes or organellar membranes [4,13,14]. Furthermore, evidence linking their dysfunction to the pathophysiology of human diseases continues to accumulate.

## 2. Genomic Information

### 2.1. SLC41A1

**Figure 1:**
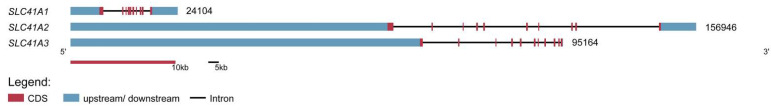
**Schematic diagram of the gene structures of the human solute carrier 41 family.** The genes are 24,104 bp, 156,946 bp, and 95,164 bp in length, respectively. Both SLC41A1 and SLC41A3 contain 10 exons, whereas SLC41A2 has 11 exons (data referenced from the website: www.ncbi.nlm.nih.gov/gene, accessed on 30 December 2025).

The gene encoding SLC41A1 is found on chromosome 1q32.1 (Table 1). Its length is 24104 bp and includes 10 exons (Figure 1). A northern blot analysis revealed that a single 5kb *SLC41A1* transcript was abundant in the heart and testis, whereas lower levels were detected in the skeletal muscle, prostate, adrenal gland, and thyroid. Hematopoietic tissues including bone marrow, lymph node, thymus, and spleen displayed only low *SLC41A1* mRNA expression levels, although lymphoid cell lines including Tom-1, BV173, Reh, and Jurkat cells were observed to express *SLC41A1* as a distinct band [12]. The *SLC41A1* gene is located at the Parkinson’s disease susceptibility locus 16 (*PARK16*); relevant information will be introduced later.

### 2.2. SLC41A2

Human *SLC41A2* is mapped to chromosome 12q23.3 and its length, 156,946 bp, is the longest among the *SLC41* family. *SLC41A2* transcripts have been identified in various organs and tissues (Table 1). The lymph nodes, stomach, lungs, testis, and skin exhibit the highest expression followed by the spleen, intestine, heart, breast, and kidney with moderate expression. The respiratory epithelia, liver, pancreas, thyroid, uterine glands, and glial cells show weak or negative expression [17].

### 2.3. SLC41A3

Human *SLC41A3* is located at chromosome 3q21.2–q21.3 (Table 1). Its length is 95,164 bp and includes 10 exons. Compared with other tissues, *SLC41A3* transcripts are the highest in testis, uterus, and ovary tissues, whereas the lowest transcripts are found in blood and liver tissue [19]. It is also differentially expressed in some cell lines, including SW480, MOLT4, MCF-7, etc. [17].

## 3. Molecular Characteristics

**Figure 2:**
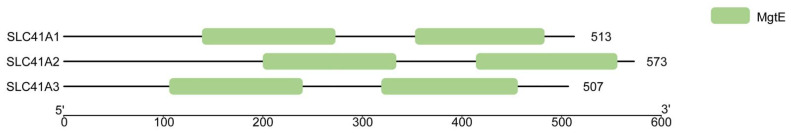
Schematic diagram of the domain structures of the human solute carrier 41 family. All three SLC41 isoforms possess two MgtE domains (data referenced from the website: www.ncbi.nlm.nih.gov/gene, accessed on 30 December 2025).

The SLC41 family of Mg^2+^ transporters expressed in vertebrates is distantly homologous to the prokaryotic MgtE Mg^2+^ transporter family. The MgtE transporter, as identified and reported by Hattori et al. [21,22], adopts a homodimeric structure with a symmetrical architecture. Its N-terminal cytosolic domains are composed of a superhelical N domain and tandemly repeated cystathionine-β-synthase (CBS) domains, which contain several Mg^2+^ binding sites. The C-terminal transmembrane domains consist of five membrane-spanning helices and form a Mg^2+^-permeable pore through homomeric dimerization. Hattori et al. suggested that the cytosolic domains may function as “intracellular Mg^2+^ sensors”, controlling the opening and closing of the pore via the “connecting helices” that link the CBS and transmembrane domains. This mechanism helps maintain intracellular Mg^2+^ concentration homeostasis. By contrast, the crystal structure of SLC41 transporters has not been fully determined. Comparing SLC41 transporters with protein databases, Wabakken et al. [12] reported that the N-terminal D1 and C-terminal D2 domains of SLC41A1 display high homology to a consensus sequence annotated Pfam01769 of MgtE. As shown in Figure 2, all three isoforms in SLC41 family possess two MgtE domains.

### 3.1. SLC41A1

In 2003, SLC41A1 was the first member of the family to be cloned and described by Wabakken et al. [12]. In both human and mouse cells, it was predicted and functionally confirmed to be an integral plasma membrane-localized protein [13,15,16]. Human SLC41A1 comprises 513 amino acids with a predicted molecular weight of approximately 56 kDa. Based on a hydropathy analysis, it is predicted to possess ten transmembrane helices (TM) and an “inside-in” configuration, i.e., both the N- and C-terminals are oriented intracellularly. While the results of independent studies have led to a consensus on the orientation of the N-terminus, the C-terminus orientation remains disputed. In an epitope-tag analysis, Mandt et al. [16] proposed an 11-TM topology with an extracellular C-terminus. Furthermore, their study showed that SLC41A1 expression and Mg^2+^ transport were regulated by Mg^2+^-dependent endosomal recycling via its N-terminal cytoplasmic domain. However, this model was not supported by a subsequent study performed by Sponder et al. [23], who advocated for the initial ten TM “inside-in” model based on data from a split-ubiquitin functional assay. Further experiments are required to conclusively determine the orientation of the C-terminus of SLC41A1.

Kolisek et al. [15] reported that SLC41A1 forms hetero-oligomeric protein complexes with high molecular mass in vivo. Most of the identified binding partners are proteins integral to the membranes of the endoplasmic reticulum (ER) and the Golgi apparatus (GA) involved in protein biosynthesis, proper folding, maturation, N-glycosylation, anterograde transport, secretion, and apoptosis regulation. SLC41A1-binding partners include the following: 3-beta-hydroxysteroid-Δ (8), Δ (7)-isomerase (emopamil-binding protein), B-cell receptor-associated protein 31, IER3IP1, PPIB, UPF0480 protein C15orf24, SPINT2, C14orf1/PEBP28, NIFIE14, YIPF6, KCP2, SLC31A2, SLC35B1, and SLC39A13. The last three are all members of the SLC superfamily and contribute to the transport of Cu^2+^ in the lysosome, sugars in the ER, and Zn^2+^ in the GA, respectively [24]. Goytain and Quamme [13] proposed N-glycosylation as a possible post-translational modification of SLC41A1 due to a putative glycosylation site at amino acid residue N-475 on the last predicted extracellular loop. Therefore, the reported identification of oligosaccharyltransferase (KCP2) as a binding partner of SLC41A1 further authenticates the proposal. An analysis of the mouse SLC41A1 amino acid sequence revealed four putative 3′,5′-cyclic monophosphate (cAMP)-dependent protein kinase A (PKA) phosphorylation sites at residues S-157, S-308, S-393, and T-508, and four possible protein kinase C (PKC) phosphorylation sites at residues T-80, T-167, T-387, and S-407. These predictions align with several functional studies (see below). In addition, SLC41A1 possesses multiple putative phosphorylation hotspots for p38MAPK, cdc2, GSK3, cdk5, DNAPK, and CKII [23]. Further studies are required to explore the involvement of these protein kinases in the regulation of SLC41A1 function.

### 3.2. SLC41A2

Sharing approximately 66% sequence identity with SLC41A1, SLC41A2 was also experimentally confirmed to be an integral plasma membrane-localized protein in both human and mouse cells [14,18]. Human SLC41A2 consists of 573 amino acids and has a predicted molecular weight of approximately 62 kDa. Based on a hydropathy analysis and the TMPred program, it is predicted to possess either 12-TM or 10-TM topologies, respectively, and an “outside-out” configuration, i.e., the N- and C-terminals are both oriented extracellularly. Nevertheless, Sahni et al. [18] reported that SLC41A2 overexpressed in DT40 chicken cells has 11 TM domains with an extracellular N-terminus and an intracellular C-terminus. Subsequently, they concluded that SLC41A2 transporters may have been aberrantly accumulated on the plasma membrane in their pilot study [18] due to overexpression [17]. They proposed the possibility that SLC41A2 is primarily functional in the membranes of the intracellular compartments. This assumption must be further tested.

### 3.3. SLC41A3

With approximately 55% similarity to SLC41A1, SLC41A3 was experimentally confirmed to be localized in the inner mitochondrial membrane in human cells [20]. Human SLC41A3 consist of 507 amino acids and has a predicted molecular weight of approximately 55 kDa. Based on the TMPred program, it is predicted to possess an 11-TM topology with an extracellular N-terminus and an intracellular C-terminus [25]. So far, no information has been available about the complex-forming abilities of SLC41A2 and SLC41A3.

## 4. Functional Studies

### 4.1. SLC41A1

Initially, the involvement of SLC41A1 in Mg^2+^-homeostasis regulation in vertebrates was authenticated. In mice fed a Mg^2+^-restricted diet, the SLC41A1 transcript level was upregulated in some tissues, including the kidney cortex and the heart [13]. Moreover, in a strain of *Salmonella enterica* with disruption in all three distinct magnesium transport systems (CorA, MgtA, and MgtB), overexpression of human SLC41A1 functionally compensated for these transporters and restored bacterial growth at low magnesium concentrations [15]. The Mg^2+^ current was observed in the *Xenopus oocytes*, heterologously expressing SLC41A1 cloned from renal mouse distal convoluted tubule cells. Currents for other divalent cations were also detected, with the following ion specificity: Mg^2+^, Sr^2+^, Fe^2+^ > Ba^2+^ > Cu^2+^ > Zn^2+^, Co^2+^ > Cd^2+^, Mn^2+^ [2,4]. Ca^2+^ did not evoke appreciable currents and was incapable of inhibiting Mg^2+^ transport. Thus, SLC41A1 should be regarded as a relatively nonselective divalent cation transporter with a preference for Mg^2+^ [13]. On the other hand, HEK293 cells overexpressing mouse SLC41A1 did not produce any measurable Mg^2+^ currents. However, with an inwardly directed Mg^2+^ gradient, they exhibited increased Mg^2+^ flux activity, which was temperature-sensitive, but not inhibitable with cobalt(III) hexamine, an Mg^2+^ channel blocker. In Mg^2+^-free solution, these cells became Mg^2+^ deficient and showed a significant loss of Mg^2+^ [15]. These data suggest that SLC41A1 functions as an Mg^2+^ carrier rather than as an Mg^2+^ channel and establish the first evidence that the protein mediates Mg^2+^ extrusion [15].

A subsequent study unveiled that SLC41A1 represents the Na^+^/Mg^2+^ exchanger (NME)—a predominant Mg^2+^-efflux system in mammalian cells [26,27]. The SLC41A1-mediated Mg^2+^ extrusion was not directly linked to anion transport, including Cl^−^ and HCO_3_^−^; however, it was strictly dependent on extracellular Na^+^ and was activated by increased intracellular Mg^2+^. Furthermore, Na^+^-dependent Mg^2+^ efflux via SLC41A1 was shown to be inhibited by the unspecific Na^+^/Mg^2+^ exchanger inhibitors imipramine and quinidine and stimulated by phosphorylation via cAMP dependent PKA [28]. Actually, several functional studies have provided evidence for the vital role of phosphorylation mediated by cAMP-activated PKA and PKC in regulating Na^+^/Mg^2+^ exchange, which is consistent with the structural predictions mentioned above. Kolisek et al. [28]. found that the hSLC41A1 protein was abundant in the phosphorylated fraction of HEK293 cells, and application of Rp-cAMP, a substance preventing PKA activation, significantly reduced SLC41A1-mediated Mg^2+^ extrusion. Insulin, a hormone that decreases cAMP production, downregulates the expression level of SLC41A1 and inhibits Mg^2+^ efflux from cells [29,30].

Based on the above research, the central controversy regarding the functional mechanism of SLC41A1 is whether it primarily acts as an Na^+^/Mg^2+^ exchanger or as an ion channel. The weight of evidence supports the former, as SLC41A1 has the following main properties of the functionally described Na^+^/Mg^2+^ exchanger. Firstly, SLC41A1 mediates a strict Na^+^-dependent Mg^2+^ efflux that is strongly activated by elevation of free intracellular Mg^2+^. Secondly, SLC41A1 is inhibited by the unspecific Na^+^/Mg^2+^ exchanger inhibitors imipramine and quinidine. Thirdly, SLC41A1 is regulated by cAMP-dependent PKA phosphorylation [28]. On the other hand, the observed currents in the SLC41A1-expressing *Xenopus oocytes* might be attributed to high-level heterologous expression and exhibit significant context-dependence [4]. Therefore, it can be concluded that SLC41A1 unquestionably represents the NME—the predominant Mg^2+^-efflux system in mammalian cells [28]. The stoichiometric ratio of Na^+^/Mg^2+^ exchange exhibits cell-type variability and may also depend on functional state. While an electroneutral 2:1 (Na^+^/Mg^2+^) ratio is most commonly reported, studies in human erythrocytes suggest alternative stoichiometries of either 3:1 or 1:1 [31]. Moreover, Arjona et al. [32] observed that SLC41A1-mediated Mg^2+^ extrusion in HEK293 cells occurs independently of extracellular Na^+^. Collectively, these findings demonstrate that a universal 2:1 exchange ratio is not consistently maintained across all cellular contexts. However, whether these discrepancies reflect true functional differences or methodological variations needs further confirmation.

The activity of NME was demonstrated to be obtained only with hydrolysable ATP or ATP analogs. Na^+^-dependent Mg^2+^ extrusion could be reduced by 90% upon depletion of ATP by treatment with FCCP or nigericin to impact an uncoupling effect on mitochondria or inhibition of mitochondrial electron transport with KCN [33,34]. Nevertheless, there is no existing evidence for modulation by direct interaction between ATP and SLC41A1. Current knowledge indicates that the NME activity is directly dependent on extracellular Na^+^ and indirectly dependent on the Na^+^/K^+^ pump, and therefore on ATP [35,36]. Inhibition of the Na^+^/K^+^ ATPase leads to increased intracellular Na^+^ concentration, which in turn inhibits NME activity [28]. Moreover, intracellular acidification to pH 6.7–6.8 induces metabolic inhibition, thereby reducing the activity of NME [34].

### 4.2. SLC41A2

In the *Xenopus oocytes* expression system, the Mg^2+^ current, as well as currents for other divalent cations, was detected, showing the following ion specificity: Mg^2+^ > Ba^2+^ > Ni^2+^ > Co^2+^ > Sr^2+^, Fe^2+^, Mn^2+^ [4]. Unlike SLC41A1, Ca^2+^ did not elicit significant currents but was capable of inhibiting Mg^2+^ transport [18]. Sahni et al. [37] reported that TRPM7-deficient DT40 cells, which originally lacked the capacity for Mg^2+^ uptake, were able to grow and proliferate in Mg^2+^-unsupplemented media upon SLC41A2 overexpression, confirming that this protein could function as a plasma membrane Mg^2+^ transporter. Consistent with this finding, Liu et al. [38] observed that expression of SLC41A2 rescued the phenotype caused by depletion of TRPM7. In fibroblasts with *TRPM7* knockdown, induction of SLC41A2 could also restore cell morphology and motility [39]. These results further support the role of SLC41A2 in maintaining cellular Mg^2+^ homeostasis in vertebrates and suggest a potential functional overlap between SLC41A2 and TRPM7 in Mg^2+^ transport mediation.

### 4.3. SLC41A3

Similar to SLC41A1 and SLC41A2, the Mg^2+^ current, as well as currents for other divalent cations, was observed for SLC41A3, with the following ion specificity: Ba^2+^ > Mg^2+^ > Zn^2+^, Ni^2+^ > Sr^2+^, Fe^2+^ > Mn^2+^ > Cu^2+^, Co^2+^ [4]. The Km value for Mg^2+^ of SLC41A3 is 1.5 mM, while the values for SLC41A1 and SLC41A2 are 0.67 mM and 0.34 mM, respectively, indicating that the Mg^2+^ affinity of SLC41A3 is the lowest in the SLC41 protein family. As both SLC41A1 and SLC41A2 mediate Mg^2+^ transport in vertebrates, SLC41A3 is also likely to play a similar functional role. Indeed, SLC41A3 has been reported to be an integral protein localized in the mitochondrial membrane that mediates Na^+^-dependent Mg^2+^ efflux from mitochondria [20]. This mitochondrial Mg^2+^ extrusion system is highly temperature-sensitive, with its capacity reduced by approximately 85% at 16 °C compared with 37 °C. Surprisingly, application of the NME inhibitor imipramine had no effect on the SLC41A3-mediated Mg^2+^ transport, a finding that requires further investigation.

## 5. Implications in Non-Tumor Diseases

### 5.1. SLC41A1

**Figure 3:**
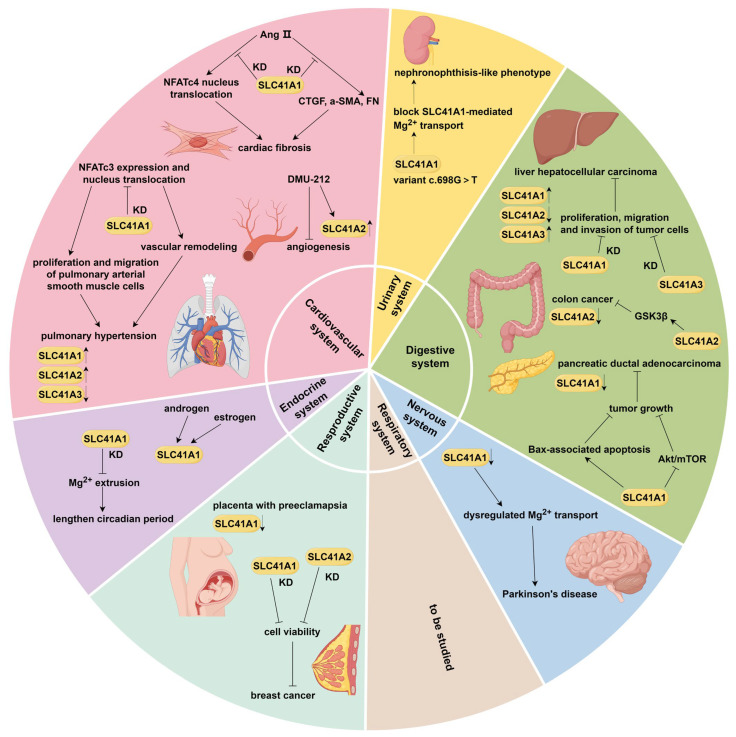
Multifaceted roles of SLC41 family members in health and diseases across multiple systems. Cardiovascular system: SLC41A1 knockdown inhibits Ang II-induced NFATc4 nuclear translocation and overexpression of CTGF, α-SMA, and FN in cardiac fibroblasts, thereby suppressing Ang II-induced cardiac fibrosis. SLC41A1 and SLC41A2 are upregulated in chronic hypoxia-induced pulmonary hypertension, whereas SLC41A3 is downregulated. SLC41A1 knockdown suppresses NFATc3 expression and nuclear translocation—a key signaling pathway promoting the proliferation and migration of hypoxic pulmonary arterial smooth muscle cells, vascular remodeling, and development of chronic hypoxia-induced PH. SLC41A2 is upregulated by DMU-212, which exhibits potent antiangiogenic effects on vascular endothelial cells. **Urinary system**: The SLC41A1 variant c.698G>T completely abolishes the Mg^2+^ transport function, leading to a nephronophthisis-like phenotype. **Digestive system**: SLC41A1 and SLC41A3 are upregulated in liver hepatocellular carcinoma, while SLC41A2 is downregulated. Knockdown of either SLC41A1 or SLC41A3 inhibits the proliferation, migration, and invasion of LIHC cells. SLC41A2 is downregulated in colon cancer and suppresses tumor progression by inhibiting ubiquitin–proteasome degradation of GSK3β, thereby upregulating GSK3β. SLC41A1 is downregulated in pancreatic ductal adenocarcinoma. Its overexpression inhibits tumor growth via Mg^2+^-dependent suppression of Akt/mTOR signaling and activation of Bax-mediated mitochondrial apoptosis. **Nervous system**: SLC41A1 is downregulated in Parkinson’s disease. Impaired Mg^2+^ transport mediated by dysregulated SLC41A1 contributes to neuronal damage and PD progression. **Respiratory system**: No studies have implicated SLC41 family members in respiratory system diseases. **Reproductive system**: SLC41A1 is significantly overexpressed in approximately 50% of preeclamptic placentas. Knockdown of either SLC41A1 or SLC41A2 reduces breast cancer cell viability. **Endocrine system**: SLC41A1 is upregulated under estrogen and androgen stimulation. SLC41A1 knockdown lengthens the circadian period by suppressing Mg^2+^ extrusion. **Abbreviations**: Ang II: angiotensin II; DMU-212: trans-3,4,5,40- tetramethoxystilbene.

A delicate balance of SLC41A1 expression is crucial, as both its upregulation and downregulation have been found to exert detrimental effects. Overexpression of SLC41A1 attenuated pro-survival signaling and reduced cell survival in multiple cell lines [40], while morpholino-mediated knockdown of *SLC41A1* resulted in severe developmental abnormalities in zebrafish [32]. These findings underscore the requirement for well-controlled levels of SLC41A1 and highlight its important prospect in diseases. Indeed, several studies have reported the association between SLC41A1 and non-tumor diseases regarding cardiovascular system, neurodegeneration, kidney, etc., as illustrated in Figure 3.

#### 5.1.1. Cardiovascular Disease

Magnesium homeostasis has long been considered to be associated with cardiovascular disease [41,42,43]. Recently, Yu et al. [44] reported that SLC41A1 may participate in angiotensin II (Ang II)-induced intracellular Mg^2+^ efflux during the acute period of stimulation in cardiac fibroblasts. Silencing *SLC41A1* inhibited Ang II-induced NFATc4 translocation to nucleus and CTGF, a-SMA, and FN overexpression in cardiac fibroblasts, thereby suppressing Ang II-induced cardiac fibrosis. In addition, chronic hypoxia- and monocrotaline-induced rat pulmonary hypertension (PH) models showed a reduction of intracellular Mg^2+^ and upregulation of SLC41A1 in pulmonary arterial smooth muscle cells (PASMCs). siRNA targeting *SLC41A1* inhibited proliferation and migration, and promoted apoptosis of PASMCs. It also suppressed the expression and nuclear translocation of NFATc3—a major signaling pathway for the proliferation and migration of hypoxic PASMCs, vascular remodeling, and development of chronic hypoxia-induced PH [45]. In a clinical study, Nestler et al. [46] reported that pregnant individuals with a high systolic blood pressure at pregnancy week also had a high expression of SLC41A1. More studies are needed to further access the underlying mechanisms.

#### 5.1.2. Neurodegenerative Disease

Satake et al. [47] discovered that the *SLC41A1* gene is located at a novel Parkinson’s disease (PD) susceptibility locus, denoted *PARK16*. In 6-hydroxyamine (6-OHDA)-induced rat models of PD, SLC41A1 expression in the striatum was downregulated during neurodegeneration, and these changes were responsive to certain concentrations of MgSO_4_. Pretreatment with MgSO_4_ before the 6-OHDA lesion induction was partially effective in preventing degeneration of dopaminergic neurons in both the substantia nigra pars compacta and the contralateral retina [48]. Consistent with this result, another study showed that SLC41A1 protein expression was also downregulated in the retinas of rat PD models, and MgSO_4_ administration not only increased the SLC41A1 expression level but protected retinal dopamine neurons [49]. These findings improve our understanding of the role of dysregulated SLC41A1-mediated Mg^2+^ transport in neuronal damage and PD progression.

Genetic analyses of this locus in PD patients identified several *SLC41A1* variants, such as p.A350V (c.1049C>T), p.K146E (c.436A>G), p.P480P (c.1440A>G), and p.R244H (c.731G>A) [50,51,52]. The conservative substitution p.A350V notably enhances Na^+^-dependent Mg^2+^ efflux and is thus considered a gain-of-function mutation. Furthermore, cells overexpressing the p.A350V variant of SLC41A1 showed insensitivity to cAMP stimulation and a decreased proliferation rate. It has been proposed that enhanced Mg^2+^ extrusion may lead to chronic intracellular Mg^2+^ deficiency in various brain regions of PD patients, potentially exacerbating neuronal damage over time [53]. By contrast, the p.R244H variant is a loss-of-function mutation that lacks detectable Mg^2+^ transport activity [52].

Additionally, genetic analyses have also revealed relationships between *SLC41A1* single nucleotide polymorphisms (SNPs) and PD. The genomic coordinates provided below are based on the GRCh38/hg38 human reference genome assembly (Table 2). For instance, in the Slovak population, the rs708727 (g.205798757G>A, synonymous variant) was associated with increased PD risk [54], whereas the rs11240569 (g.205810103G>A,C, synonymous variant) appeared to reduce risk for PD in both the Chinese and Iranian population [55,56]. Similarly, the variant rs823156 (g.205795512G>A,C,T, intron variant) was associated with reduced PD risk in the Ashkenazi Jewish population [57]. A bioinformatic analysis suggested that rs823156 may act as a noncoding variant of SLC41A1 affecting PD risk by altering transcription factor binding affinity, providing new mechanistic insights that warrant further investigation [57].

#### 5.1.3. Others

Another variant, p.G223V (c.698G>T), in the *SLC41A1* gene was found to cause a nephronophthisis-like phenotype (NPHLP) [60]. This mutation leads to the skipping of exon 6 of SLC41A1, resulting in an in-frame deletion of a transmembrane helix. Further functional studies have demonstrated that this mutation completely blocks the Mg^2+^ transport function of SLC41A1. Moreover, in normal human kidney tissue, endogenous SLC41A1 is specifically expressed in the renal tubules located at the corticomedullary boundary—a region consistent with the site of cystogenesis observed in NPHP. Collectively, these findings suggest that disrupted Mg^2+^ homeostasis caused by SLC41A1 mutation may lead to tubular defects that result in a nephronophthisis-like phenotype.

SLC41A1 was also found to be significantly overexpressed in approximately half of the placenta with preeclampsia, indicating a direct contribution of enhanced Mg^2+^ transport activity in the development of preeclampsia [61]. Data from the Women’s Health Initiative-SNP Health Association Resource study revealed that the *SLC41A1* polymorphism rs823154 (g.205793278C>T, intron variant) is associated with an increased risk of type 2 diabetes (T2D) among Hispanic-American women with low magnesium intake [58]. In addition, Feeney et al. [62] reported a link between SLC41A1 and circadian rhythm regulation. Both inhibition of the Na^+^/Mg^2+^ exchanger with quinidine and the siRNA-mediated knockdown of *SLC41A1* resulted in a significant Mg^2+^-dependent lengthening of the circadian period by suppressing Mg^2+^ extrusion. It has also been reported that SLC41A1 expression is upregulated under the conditions of estrogen and androgen stimulation [63,64].

### 5.2. SLC41A2

Far fewer studies have implicated SLC41A2 in non-tumor diseases compared to SLC41A1. The *SLC41A2* polymorphism rs10861279 (g.104821412A>C,G, intron variant) as well as a 2-SNP-haplotype, rs12582312 (g.104821402C>T, intron variant)-rs10861279 has been associated with an increased risk of T2D among Hispanic-American women [58]. SLC41A2 is also upregulated by trans-3,4,5,40-tetramethoxystilbene (DMU-212), which possesses potent proapoptosis and antiangiogenesis effects on vascular endothelial cells [65]. Consistent with the finding of SLC41A1, chronic hypoxia- and monocrotaline-induced rat PH models showed reduced intracellular Mg^2+^ levels and upregulation of SLC41A2 in PASMCs. The siRNA-mediated knockdown of *SLC41A2* inhibited proliferation and migration, and promoted apoptosis of PASMCs. However, it had no effect on the expression of NFATc3 [45]. Further studies are needed to elucidate the underlying mechanisms.

### 5.3. SLC41A3

In contrast to SLC41A1 and SLC41A2, SLC41A3 was downregulated in the PASMCs of chronic hypoxia- and monocrotaline-induced rat PH models, and its overexpression caused similar effects to siRNA targeting *SLC41A1* and *SLC41A2* [45]. Given that SLC41A3 shares only approximately 55% sequence identity with SLC41A1, their roles in disease may differ. Tur et al. [66] reported that deletion of Kvβ2 attenuates isoproterenol-induced cardiac injury possibly through SLC41A3 downregulation. The rs71327750 (g.126096213G>T, intron variant) of *SLC41A3* showed evidence of association with dental caries in both the adult and pediatric cohorts of the GLIDE consortium [59]. Additionally, the whole-genome sequencing of dogs with stomatocytosis identified a novel genetic variant in *SLC41A3*, namely chr20:195,027 in the affected Australian Cattle Dog. This variant is predicted to be deleterious to SLC41A3 function, suggesting that it may act as a novel causal driver or modifier of the canine stomatocytosis phenotype [67].

## 6. Implications in Tumor Diseases

### 6.1. SLC41A1

There are a limited number of studies indicating the implications of SLC41A1 in tumor diseases. Uddin et al. [68,69] found that the delivery of siRNA against SLC41A1 resulted in the reduction of cell viability and tumor volume of breast cancer, suggesting that SLC41A1 may be a promising therapeutic target for siRNA-mediated knockdown in breast cancer. Additionally, SLC41A1 was downregulated in pancreatic ductal adenocarcinoma (PDAC). Its overexpression suppressed orthotopic tumor growth through Mg^2+^-dependent Akt/mTOR inhibition and Bax-associated mitochondrial apoptosis [70]. SLC41A1 was also found to upregulate in liver hepatocellular carcinoma (LIHC), which was confirmed by immunostaining of LIHC patients. Cellular experiments demonstrated that the knockdown of *SLC41A1* suppressed the proliferation, migration, and invasion of LIHC cells, whereas its overexpression exerted tumor-promoting effects [71]. Collectively, these studies identify SLC41A1 as a novel diagnostic biomarker and therapeutic target for breast cancer, PDAC, and LIHC.

**Figure 4:**
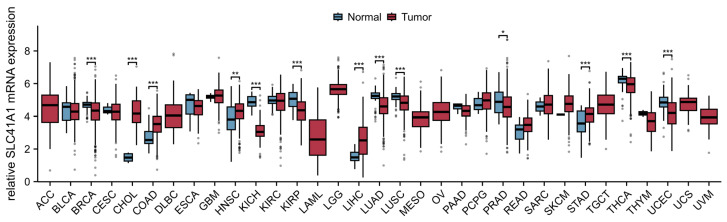
**SLC41A1 is dysregulated in pan-cancer analysis**. SLC41A1 expression is significantly upregulated in tumors such as CHOL, COAD, HNSC, LIHC, and STAD compared to normal tissues. By contrast, it is downregulated in BRCA, KICH, KIRP, LUAD, LUSC, PRAD, THCA, and UCEC. No significant difference in SLC41A1 expression was observed in ACC, BLCA, CESC, DLBC, ESCA, GBM, KIRC, LAML, LGG, MESO, OV, PAAD, PCPG, READ, SARC, SKCM, TGCT, THYM, UCS, or UVM. Abbreviations: ACC, adrenocortical carcinoma; BLCA, bladder urothelial carcinoma; BRCA, breast invasive carcinoma; CESC, cervical squamous cell carcinoma and endocervical adenocarcinoma; CHOL, cholangiocarcinoma; COAD, colon adenocarcinoma; DLBC, lymphoid neoplasm diffuse large B-cell lymphoma; ESCA, esophageal carcinoma; GBM, glioblastoma multiforme; HNSC, head and neck squamous cell carcinoma; KICH, kidney chromophobe; KIRC, kidney renal clear cell carcinoma; KIRP, kidney renal papillary cell carcinoma; LAML, acute myeloid leukemia; LGG, brain lower grade glioma; LIHC, liver hepatocellular carcinoma; LUAD, lung adenocarcinoma; LUSC, lung squamous cell carcinoma; MESO, mesothelioma; OV, ovarian serous cystadenocarcinoma; PAAD, pancreatic adenocarcinoma; PCPG, pheochromocytoma and paraganglioma; PRAD, prostate adenocarcinoma; READ, rectum adenocarcinoma; SARC, sarcoma; SKCM, skin cutaneous melanoma; STAD, stomach adenocarcinoma; TGCT, testicular germ cell tumors; THCA, thyroid carcinoma; THYM, thymoma; UCEC, uterine corpus endometrial carcinoma; UCS, uterine carcinosarcoma; UVM, uveal melanoma. (Data obtained from The Cancer Genome Atlas [TCGA] database. * *p* < 0.05, ** *p* < 0.01, *** *p* < 0.001. https://www.xiantaozi.com/products/apply/c0b6febb-52dd-4525-970a-61bbe9e263ff/analyse/4c894ac7-35f8-4f44-aecc-85643fa85ca1) (accessed date: 29 December 2025).

**Figure 5:**
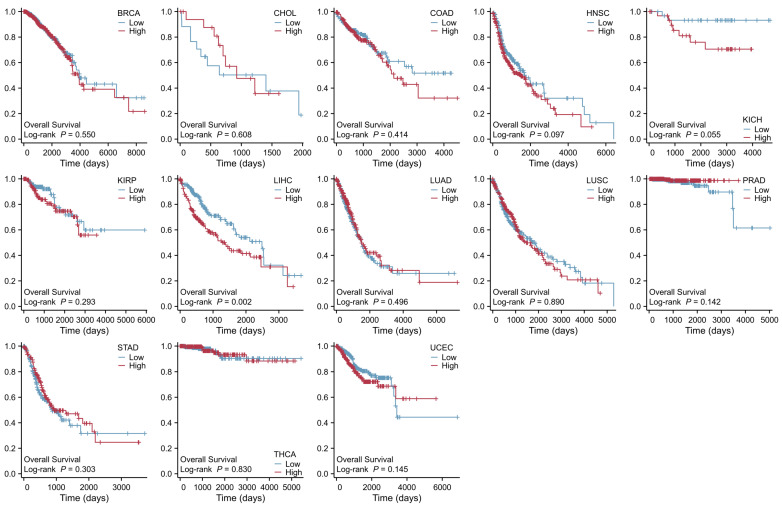
**Prognostic value of SLC41A1 expression in various cancers.** High expression of SLC41A1 is associated with poor prognosis in patients with LIHC. By contrast, no significant association was observed between SLC41A1 expression and patient survival in BRCA, CHOL, COAD, HNSC, KICH, KIRP, LUAD, LUSC, PRAD, STAD, THCA, or UCEC. (Data obtained from The Cancer Genome Atlas [TCGA] database: https://www.xiantaozi.com/products/apply/c0b6febb-52dd-4525-970a-61bbe9e263ff/analyse/fc4754b7-d0fa-44af-9b0d-fa4e3fe1b7b3) (accessed date: 29 December 2025).

The role of SLC41A1 in other tumor diseases has not yet been reported. As shown in Figure 4, we analyzed the data from the TCGA database (https://portal.gdc.cancer.gov/, accessed on 30 December 2025) and found that the SLC41A1 level is significantly increased in tumors, including cholangiocarcinoma (CHOL), colon adenocarcinoma (COAD), head and neck squamous cell carcinoma (HNSC), LIHC, and stomach adenocarcinoma (STAD), while it is decreased in breast invasive carcinoma (BRCA), kidney chromophobe (KICH), kidney renal papillary cell carcinoma (KIRP), lung adenocarcinoma (LUAD), lung squamous cell carcinoma (LUSC), prostate adenocarcinoma (PRAD), thyroid carcinoma (THCA), and uterine corpus endometrial carcinoma (UCEC) compared with non-tumor tissue. Additionally, the results exhibited that an increased SLC41A1 level is associated with poor prognosis and is known to affect the survival of patients suffering LIHC, consistent with the above-mentioned study, as shown in Figure 5.

### 6.2. SLC41A2

Recent studies have reported several pathological roles of SLC41A2 in breast cancer, hepatitis B virus (HBV)-induced LIHC, and colon cancer. Uddin et al. [68] observed that both MCF-7 and 4T1 breast cancer cells showed a significant decrease in cell viability after transfection with functionally validated siRNA targeting *SLC41A2*, and the effect was more pronounced than that seen with siRNA targeting other genes of magnesium transporters, *SLC41A1* and *MAGT1*. A comprehensive bioinformatics study systematically identified 573 differentially expressed miRNAs across the spectrum of HBV-induced liver pathologies and revealed several novel miRNA–mRNA regulatory axes in LIHC. A key finding was the identification of hsa-miR-522-5p as an upregulated miRNA that may target and downregulate *SLC41A2*, implying a previously unrecognized mechanism in HBV–LIHC pathogenesis and a therapeutic target [72]. In colon cancer, SLC41A2 exhibited a lower expression level when compared to normal tissues. Functional studies have demonstrated that overexpression of SLC41A2 significantly decreased the proliferation, migration, and invasion of colon cancer cells, along with increased apoptosis. Mechanistically, it inhibits ubiquitin–proteasome degradation of GSK3β, leading to GSK3β upregulation and consequent suppression of colon cancer progression [73].

**Figure 6:**
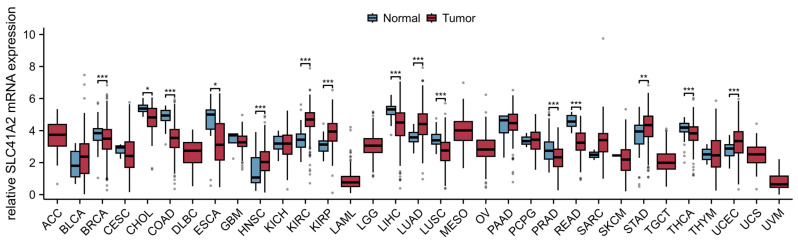
**SLC41A2 is dysregulated in pan-cancer analysis.** SLC41A2 expression is significantly upregulated in tumors including HNSC, KIRC, KIRP, LUAD, STAD, and UCEC. By contrast, it is downregulated in BRCA, CHOL, COAD, ESCA, LIHC, LUSC, PRAD, READ, and THCA. No significant difference in expression was observed in ACC, BLCA, CESC, DLBC, GBM, KICH, LAML, LGG, MESO, OV, PAAD, PCPG, SARC, SKCM, TGCT, THYM, UCS, or UVM (Data obtained from The Cancer Genome Atlas [TCGA] database. * *p* < 0.05, ** *p* < 0.01, *** *p* < 0.001. https://www.xiantaozi.com/products/apply/c0b6febb-52dd-4525-970a-61bbe9e263ff/analyse/4c894ac7-35f8-4f44-aecc-85643fa85ca1) (accessed date: 29 December 2025).

**Figure 7:**
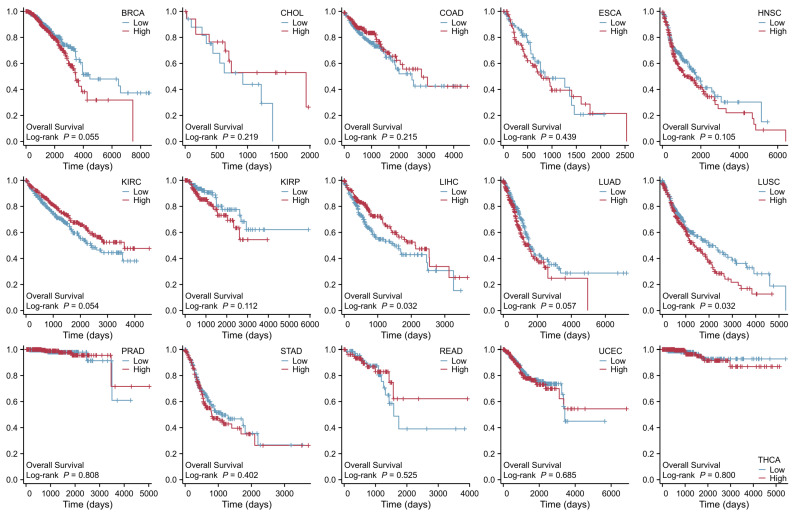
**Prognostic value of SLC41A2 expression in various cancers.** Low SLC41A2 expression was associated with poor prognosis in LIHC, while its high expression was associated with reduced survival in LUSC. No significant association was observed in several other cancers, including BRCA, CHOL, COAD, ESCA, HNSC, KIRC, KIRP, LUAD, PRAD, READ, STAD, THCA, and UCEC. (Data obtained from The Cancer Genome Atlas [TCGA] database: https://www.xiantaozi.com/products/apply/c0b6febb-52dd-4525-970a-61bbe9e263ff/analyse/fc4754b7-d0fa-44af-9b0d-fa4e3fe1b7b3) (accessed date: 29 December 2025).

As shown in Figure 6, we also analyzed the data of SLC41A2 in pan-cancer. The results indicated that SLC41A2 expression is increased in HNSC, kidney renal clear cell carcinoma (KIRC), KIRP, LUAD, STAD, and UCEC, but is decreased in BRCA, CHOL, COAD, esophageal carcinoma (ESCA), LIHC, LUSC, PRAD, rectum adenocarcinoma (READ), and THCA. Patients with low SLC41A2 expression showed poor prognosis in LIHC, whereas elevated expression SLC41A2 is inversely correlated with the survival of patients with LUSC, as shown in Figure 7.

### 6.3. SLC41A3

To date, a growing body of evidence has established an association between SLC41A3 and LIHC. Compared to normal tissue, LIHC exhibits significantly lower DNA methylation levels at the *SLC41A3* locus, which may account for its overexpression in tumor tissues [19]. Functional studies have shown that silencing *SLC41A3* inhibits the proliferation, migration, and invasion of LIHC cells in vitro [74]. Furthermore, overexpression of SLC41A3 is also linked to shorter survival times and reduced survival rates in LIHC patients [75,76]. Thus, SLC41A3 may serve as both a prognostic biomarker and a potential therapeutic target in LIHC.

Furthermore, a comprehensive bioinformatics study indicated the role of SLC41A3 in pan-cancer. The results from all three databases exhibited high SLC41A3 expressions in bladder urothelial carcinoma (BLCA), CHOL, COAD, LIHC, LUSC, and READ, but low SLC41A3 expression in BRCA, KICH, KIRC, UCEC, and THCA when compared with non-tumor tissue. The results from two databases indicated that SLC41A3 expression was also significantly elevated in ESCA, HNSC, and STAD; however, these differences were not statistically significant in the other dataset. Based on the TCGA database, the results indicated that increased SLC41A3 expression predicted better prognosis in adenoid cystic carcinoma (ACC), lymphoid neoplasm diffuse large B-cell lymphoma (DLBC), KIRP, KIRC, pheochromocytoma and paraganglioma (PCPG), thymoma (THYM), and uveal melanoma (UVM); nevertheless, patients with low SLC41A3 expression showed remarkably good clinical outcome in LIHC and ovarian serous cystadenocarcinoma (OV), while there was no prognostic relevance of SLC41A3 expression in some common tumors, including BRCA, LUAD, and brain lower grade glioma (LGG) [19].

### 6.4. Mechanistic Summary 

Based on the studies presented above, the roles of SLC41 family members in various cancers can be synthesized according to their primary molecular mechanisms.

#### 6.4.1. Modulation of Cell Proliferation and Apoptosis

SLC41A1 and SLC41A3 exerts a tumor-promoting effect in LIHC by driving cell proliferation, migration, and invasion. In PDAC, SLC41A1 inhibits tumor growth partly via activation of Bax-mediated mitochondrial apoptosis. SLC41A2 acts as a tumor suppressor in colon cancer, where its overexpression significantly inhibits cancer cell proliferation, migration, and invasion.

#### 6.4.2. Regulation of Key Signaling Pathways

SLC41A1 suppresses tumor growth through the inhibition of the Mg^2+^-dependent Akt/mTOR pathway in PDAC. SLC41A2 inhibits colon cancer progression by suppressing the ubiquitin–proteasome degradation of GSK3β, leading to GSK3β protein upregulation. Its expression in HBV–LIHC is potentially regulated by the miRNA hsa-miR-522-5p. SLC41A3 overexpression in LIHC is associated with significantly lower DNA methylation levels at its gene locus, suggesting epigenetic dysregulation as a key mechanism for its upregulation.

## 7. Conclusions

This review synthesizes current knowledge of the SLC41 Mg^2+^ transporters, including their genomic information, molecular characteristics, functions, and multifaceted roles in diseases. SLC41A1 functions as the Na^+^/Mg^2+^ exchanger, a predominant Mg^2+^-efflux system, with its dysregulation implicated in conditions ranging from Parkinson’s disease to pulmonary hypertension and various cancers. SLC41A2 mediates Mg^2+^ fluxes, but it is currently unclear whether it operates at the plasma membrane or at organellar membranes. Localized to the mitochondrial inner membrane, SLC41A3 facilitates Na^+^-dependent Mg^2+^ efflux from mitochondria. Despite these advances, several challenges persist, outlining clear directions for future research.

### 7.1. Underlying Mechanisms

The precise molecular structure of SLC41 transporters and the detailed mechanisms underlying their roles in specific diseases, are still poorly understood. Notably, a consensus on the membrane topology for all three members is lacking, and the cellular localization of SLC41A2 has not been definitively established. Furthermore, in contrast to SLC41A1, SLC41A2 expression is downregulated in LIHC, and this downregulation correlates with poor patient prognosis. Elucidating these mechanisms will require comprehensive in vivo and in vitro functional studies. The relationship between their Mg^2+^ transport function and other signaling pathways, particularly in Parkinson’s disease, requires further investigation.

### 7.2. Pharmacological Targeting: Current Status and Future Directions

#### 7.2.1. Current Challenges in SLC41 Pharmacology

Despite the growing recognition of SLC41 transporters as potential therapeutic targets, the development of specific pharmacological modulators remains in its infancy. No selective small-molecule inhibitors or activators targeting any SLC41 family member have been reported to date, and the field currently lacks high-affinity chemical probes for functional validation. This pharmacological void presents a significant bottleneck in translating basic research findings into therapeutic applications. Several fundamental challenges contribute to this gap. Firstly, the incomplete resolution of SLC41 transporter structures, particularly the ongoing controversy regarding SLC41A1’s membrane topology (10-TM vs. 11-TM models) [4], severely hampers rational drug design. Secondly, current “inhibitors” cited in the literature, such as imipramine and quinidine, exhibit broad polypharmacology and lack selectivity for SLC41 transporters [28]. Thirdly, the functional overlap between SLC41 isoforms and other Mg^2+^ transporters (e.g., TRPM6/7, MagT1) creates compensatory mechanisms that may mask the phenotypic effects of pharmacological inhibition. Moreover, SLC41 transporters exhibit tissue-specific expression and differential regulation, suggesting that their pharmacological targeting may require tissue-selective approaches to minimize systemic side effects.

#### 7.2.2. Future Research Directions in SLC41 Pharmacology

To overcome these challenges and to advance the therapeutic targeting of SLC41 transporters, several strategic approaches should be prioritized. Firstly, Cryo-EM studies of SLC41 transporters in different conformational states are urgently needed to resolve topological ambiguities and identify potential ligand-binding sites. Secondly, chemical probes should be developed by high-throughput screening campaigns to identify lead compounds with improved selectivity. Thirdly, isoform-selective inhibitors should be developed to dissect the individual contributions of SLC41A1, A2, and A3 in different physiological contexts. Conditional knockout models combined with pharmacological inhibition could help distinguish on-target effects from compensatory mechanisms. Finally, given the divergent roles of SLC41 isoforms in different pathologies (e.g., SLC41A1 in Parkinson’s disease vs. cancer), disease-specific targeting strategies should be explored; furthermore, combination therapies with existing drugs (e.g., Mg^2+^ supplementation in Parkinson’s disease) could be evaluated once selective modulators become available.

### 7.3. Integrative Studies

Future research would benefit from employing more sophisticated models, such as tissue-specific knockout mice and 3D organoid cultures, to validate the pathophysiological roles of SLC41 transporters in vivo and explore their potential as diagnostic biomarkers or therapeutic targets across a broader range of diseases.

In conclusion, SLC41 Mg^2+^ transporters represent a crucial node linking magnesium homeostasis to human diseases, targeting them has emerged as a promising therapeutic strategy, yet overcoming the existing challenges will be essential for realizing this promise.

## Figures and Tables

**Table 1:** Genomic information and molecular characteristics of SLC41 transporters.

Name	SLC41A1	SLC41A2	SLC41A3
Gene localization	1q32.1	12q23.3	3q21.2–q21.3
Gene length	24,104	156,946	95,164
Exon	10	11	10
Tissue distribution	High expression: heart and testis; moderate expression: skeletal muscle, prostate, adrenal gland, and thyroid; low expression: hematopoietic tissues bone marrow, lymph node, thymus, and spleen	High expression: lymph nodes, stomach, lungs, testis, and skin; moderate expression: spleen, intestine, heart, breast, and kidney; low expression: respiratory epithelia, liver, pancreas, thyroid, uterine glands and glial cells	High expression: testis, uterus, and ovary tissues; low expression: blood and liver tissue
Amino acids	513	573	507
Membrane	Plasma membrane	Plasma membrane or organellar membrane	Mitochondrial membrane
Km value	0.67 mM	0.34 mM	1.5 mM
Function	Na^+^-dependent Mg^2+^ efflux	Mg^2+^ fluxes	Na^+^-dependent Mg^2+^ efflux
Reference	[2,4,12,13,15,16]	[4,14,17,18]	[4,17,19,20]

**Table 2:** Relationships between SLC41s single nucleotide polymorphism and diseases.

Protein	Disease	Single Nucleotide Polymorphism	Impact	References
SLC41A1	Parkinson’s disease	rs708727	increase	[54]
		rs11240569	decrease	[55,56]
		rs823156	decrease	[57]
	type 2 diabetes	rs823154	increase	[58]
SLC41A2	type 2 diabetes	rs10861279, rs12582312-rs10861279	increase	[58]
SLC41A3	dental caries	rs71327750	increase	[59]

## Data Availability

Datasets analyzed during the current study are publicly available in the XENA TCGA GTEx-ALL database (https://xenabrowser.net/datapages/, accessed on 30 December 2025) and Kaplan–Meier survival analyses (https://kmplot.com/analysis/, accessed on 30 December 2025).

## Abbreviations

The following abbreviations are used in this manuscript:

Mg^2+^	Magnesium ion
SLC41	Solute Carrier Family 41
MgtE	Magnesium Transporter E
TRPM6/7	Transient Receptor Potential Cation Channel Subfamily M Member 6/7
NIPA	Nonimprinted in Prader–Willi/Angelman Syndrome
CNNM1-4	Cyclin and CBS Domain Divalent Metal Cation Transport Mediator 1-4
MagT1	Magnesium Transporter 1
MRS2	Mitochondrial RNA Splicing 2
PARK16	Parkinson’s Disease Susceptibility Locus 16
CBS	Cystathionine-β-synthase
TM	Transmembrane Helix
ER	Endoplasmic Reticulum
GA	Golgi Apparatus
cAMP	3′,5′-Cyclic Monophosphate
PKA	Protein Kinase A
PKC	Protein Kinase C
NME	Na^+^/Mg^2+^ Exchanger
ATP	Adenosine Triphosphate
FCCP	Carbonyl Cyanide-4-(trifluoromethoxy)phenylhydrazone
KCN	Potassium Cyanide
Ang II	Angiotensin II
NFATc4	Nuclear Factor of Activated T-cells, Cytoplasmic 4
CTGF	Connective Tissue Growth Factor
α-SMA	Alpha-Smooth Muscle Actin
FN	Fibronectin
PH	Pulmonary Hypertension
PASMCs	Pulmonary Arterial Smooth Muscle Cells
PD	Parkinson’s Disease
6-OHDA	6-Hydroxydopamine
SNP	Single Nucleotide Polymorphism
NPHLP	Nephronophthisis-like Phenotype
T2D	Type 2 Diabetes
siRNA	Small Interfering RNA
PDAC	Pancreatic Ductal Adenocarcinoma
LIHC	Liver Hepatocellular Carcinoma
TCGA	The Cancer Genome Atlas
CHOL	Cholangiocarcinoma
COAD	Colon Adenocarcinoma
HNSC	Head and Neck Squamous Cell Carcinoma
STAD	Stomach Adenocarcinoma
BRCA	Breast Invasive Carcinoma
KICH	Kidney Chromophobe
KIRP	Kidney Renal Papillary Cell Carcinoma
LUAD	Lung Adenocarcinoma
LUSC	Lung Squamous Cell Carcinoma
PRAD	Prostate Adenocarcinoma
THCA	Thyroid Carcinoma
UCEC	Uterine Corpus Endometrial Carcinoma
HBV	Hepatitis B Virus
KIRC	Kidney Renal Clear Cell Carcinoma
ESCA	Esophageal Carcinoma
READ	Rectum Adenocarcinoma
BLCA	Bladder Urothelial Carcinoma
ACC	Adenoid Cystic Carcinoma
DLBC	Lymphoid Neoplasm Diffuse Large B-cell Lymphoma
PCPG	Pheochromocytoma and Paraganglioma
THYM	Thymoma
UVM	Uveal Melanoma
OV	Ovarian Serous Cystadenocarcinoma
LGG	Brain Lower Grade Glioma
